# Neuronal Loss of the Glutamate Transporter GLT-1 Promotes Excitotoxic Injury in the Hippocampus

**DOI:** 10.3389/fncel.2021.788262

**Published:** 2021-12-29

**Authors:** Theresa S. Rimmele, Shaomin Li, Jens Velde Andersen, Emil W. Westi, Alexander Rotenberg, Jianlin Wang, Blanca Irene Aldana, Dennis J. Selkoe, Chiye J. Aoki, Chris G. Dulla, Paul Allen Rosenberg

**Affiliations:** ^1^Department of Neurology and the F. M. Kirby Neurobiology Center, Boston Children’s Hospital, Boston, MA, United States; ^2^Ann Romney Center for Neurologic Diseases, Department of Neurology, Brigham and Women’s Hospital and Harvard Medical School, Boston, MA, United States; ^3^Department of Drug Design and Pharmacology, University of Copenhagen, Copenhagen, Denmark; ^4^Program in Neuroscience, Harvard Medical School, Boston, MA, United States; ^5^Center for Neural Science, New York University, NY, United States; ^6^Neuroscience Institute NYU Langone Medical Center, NY, United States; ^7^Department of Neuroscience, Tufts University School of Medicine, Boston, MA, United States

**Keywords:** homeostasis, excitotoxicity glutamatergic, aging, neurodegeneration, mitochondria, Alzheimer’s disease, repair

## Abstract

GLT-1, the major glutamate transporter in the mammalian central nervous system, is expressed in presynaptic terminals that use glutamate as a neurotransmitter, in addition to astrocytes. It is widely assumed that glutamate homeostasis is regulated primarily by glutamate transporters expressed in astrocytes, leaving the function of GLT-1 in neurons relatively unexplored. We generated conditional GLT-1 knockout (KO) mouse lines to understand the cell-specific functions of GLT-1. We found that stimulus-evoked field extracellular postsynaptic potentials (fEPSPs) recorded in the CA1 region of the hippocampus were normal in the astrocytic GLT-1 KO but were reduced and often absent in the neuronal GLT-1 KO at 40 weeks. The failure of fEPSP generation in the neuronal GLT-1 KO was also observed in slices from 20 weeks old mice but not consistently from 10 weeks old mice. Using an extracellular FRET-based glutamate sensor, we found no difference in stimulus-evoked glutamate accumulation in the neuronal GLT-1 KO, suggesting a postsynaptic cause of the transmission failure. We hypothesized that excitotoxicity underlies the failure of functional recovery of slices from the neuronal GLT-1 KO. Consistent with this hypothesis, the non-competitive NMDA receptor antagonist MK801, when present in the ACSF during the recovery period following cutting of slices, promoted full restoration of fEPSP generation. The inclusion of an enzymatic glutamate scavenging system in the ACSF conferred partial protection. Excitotoxicity might be due to excess release or accumulation of excitatory amino acids, or to metabolic perturbation resulting in increased vulnerability to NMDA receptor activation. Previous studies have demonstrated a defect in the utilization of glutamate by synaptic mitochondria and aspartate production in the synGLT-1 KO *in vivo*, and we found evidence for similar metabolic perturbations in the slice preparation. In addition, mitochondrial cristae density was higher in synaptic mitochondria in the CA1 region in 20–25 weeks old synGLT-1 KO mice in the CA1 region, suggesting compensation for loss of axon terminal GLT-1 by increased mitochondrial efficiency. These data suggest that GLT-1 expressed in presynaptic terminals serves an important role in the regulation of vulnerability to excitotoxicity, and this regulation may be related to the metabolic role of GLT-1 expressed in glutamatergic axon terminals.

## Introduction

Most synapses have a mechanism for neurotransmitter reuptake in the presynaptic terminal, and the demonstration of a high-affinity specific uptake system for glutamate in purified synaptosomes provided important biochemical evidence that this amino acid is a neurotransmitter (Logan and Snyder, 1971; Bennett et al., 1972; Rimmele and Rosenberg, 2016). In excitatory presynaptic terminals, the high-affinity glutamate transporter GLT-1 is expressed in many but not all synapses (Chen et al., 2004; Berger et al., 2005; Furness et al., 2008). In the hippocampus, 80–90% of GLT-1 is found in glial cells and 5–10% in axon terminals (Furness et al., 2008), and a consensus has arisen that glutamate clearance is primarily if not exclusively accomplished by astrocytic GLT-1 (Bergles et al., 1999; Danbolt, 2001; Tzingounis and Wadiche, 2007). Glutamate homeostasis (Schousboe and Hertz, 1981; Ottersen et al., 1996; Schousboe et al., 1997; Takahashi et al., 1997; Bezzi et al., 1999; Kalivas, 2009) is important for the survival of neurons in the CNS in the face of the constant threat of excitotoxicity due to excess or abnormal activation of glutamate receptors (Lipton and Rosenberg, 1994). In addition, glutamate homeostasis has emerged as a critical determinant of important neurobiological phenomena, including pain (Inquimbert et al., 2012, 2018), addiction (Fischer et al., 2020), mental illness (Hu et al., 2015; O’Donovan et al., 2015; Parkin et al., 2018, 2020) plasticity (Levenson et al., 2000a,b, 2002; Collado et al., 2007, 2009), and chronic neurodegeneration, in particular, in Alzheimer’s disease (Li et al., 2009; Zott et al., 2019). In general, it has been assumed that these multiple roles for GLT-1 are implemented by GLT-1 expressed in astrocytes, whereas the functions of the small amount of GLT-1 expressed in axon terminals remain largely unknown.

Recent studies have shown that GLT-1 expressed in axon terminals may serve an important metabolic role (McNair et al., 2019, 2020; Andersen et al., 2021a), although the functional importance of this metabolic involvement of GLT-1 and the consequences of its perturbation or disruption have not been explored. To pursue the cell-type specific functions of GLT-1, we generated a conditional GLT-1 knockout and mouse lines using Cre/lox technology to: *(i)* inactivate the GLT-1 gene in astrocytes, by a tamoxifen-inducible glial fibrillary acidic protein (GFAP) driver (Casper et al., 2007) of Cre-recombinase expression (gfapGLT-1 KO), and *(ii)* using a synapsin 1 driver of Cre-recombinase expression (Zhu et al., 2001) to inactivate GLT-1 specifically in neurons (synGLT-1 KO; Petr et al., 2015). Like the pan GLT-1 KO (Tanaka et al., 1997), gfapGLT-1 KO mice have intractable seizures and a shortened life span (Petr et al., 2015). The synGLT-1 KO, in contrast, is behaviorally normal up to 12 months of age (Petr et al., 2015; Sharma et al., 2019) but has been found to have a defect in the performance of the Morris Water Maze at 18 months of age suggesting impaired hippocampal memory formation (Vorhees and Williams, 2006; Sharma et al., 2019).

Neurons in the CA3 region of the hippocampus express GLT-1 mRNA at the highest levels found in neurons anywhere in the brain (Torp et al., 1994, 1997; Schmitt et al., 1996; Berger and Hediger, 1998; Berger et al., 2005). If GLT-1 expressed in axon terminals has a role in synaptic transmission it should be manifest at the CA3 to CA1 synapse that can be conveniently studied using the hippocampal slice preparation. The hippocampal slice preparation is also considered to be a model for brain injury and repair, in that during the preparation of slices the brain is subjected to both ischemic and traumatic injury and after an initial period of electrical silence undergoes repair processes that restore synaptic transmission (Kirov et al., 1999; Fiala et al., 2003; Buskila et al., 2014; Rae and Balcar, 2014; Frenguelli, 2019). Accordingly, the slice preparation has been used as a model in which it is possible to study how genetic and other manipulations affect processes associated with repair and recovery from injury (Hossmann, 2008; Hall and Frenguelli, 2018; Frenguelli, 2019; Frenguelli and Dale, 2020). In this study, we took advantage of the hippocampal slice preparation to test whether the inactivation of GLT-1 in neurons affects synaptic function and synaptic health. In fact, we found that the generation of field excitatory postsynaptic potentials was compromised in slices from synGLT-1 KO animals 20 weeks and older, and this compromise appeared to be due to excitotoxic injury. Remarkably, fEPSP generation in gfapGLT-1 KO animals was normal at least through 40 weeks of age. These findings suggest that, despite its low level of expression, GLT-1 expressed in axon terminals serves an important role in regulating vulnerability to excitotoxicity.

## Materials and Methods

### Mice

Male conditional GLT-1 knock-out mice were obtained from the founding colony at Boston Children’s Hospital (Slc1A2^tm1.1Pros^; MGI: 5752263; Petr et al., 2015). Neuronal GLT-1 knockout mice were generated in which the GLT-1 gene was inactivated in neurons by expression of synapsin-Cre as described previously (GLT-1^flox/flox^; synapsin-Cre; Petr et al., 2015; Fischer et al., 2018), and littermate controls with normal GLT-1 function (GLT-1^flox/flox^). These are referred to in the current article as synGLT-1 KO and wild-type littermate controls, respectively. In order to determine that our observations were not due to Cre-recombinase expression, *per se* (Harno et al., 2013), we generated and used synapsin-Cre control mice. Male mice with a tamoxifen-inducible astrocyte-specific knock-out of GLT-1 (GLT-1^Δ/Δ^;GFAP-Cre ERT2) are referred to as gfapGLT-1 KO and were generated using the hGFAP-CreER^T2^ driver (Casper et al., 2007) as described previously (Petr et al., 2015). Pups from an entire litter were treated daily with tamoxifen (T5648, Sigma-Aldrich; 33 mg/kg, i.p. or oral gavage in sunflower oil) starting from P5 and for 4–5 consecutive days (Ganat et al., 2006). Tamoxifen solutions were made fresh for a given litter and never frozen. Experiments were conducted on adult male mice, synGLT-1 KO experiments were performed with three age groups, 10–12 weeks (referred to as 10 weeks throughout the manuscript), 18–21 weeks (referred to as 20 weeks throughout the manuscript), and 30–40 weeks of age, using age-matched littermates as controls; gfapGLT-1 KO experiments were performed with 24–40 weeks old mice and littermate controls. For the electron microscopic study, a single age group of 22–24 weeks was used. Animals were housed in a temperature-controlled room on a 12-h light/12-h dark cycle and had ad libitum access to food and water.

Mice were maintained on a 129S4/SvJaeJ (JAX Stock No. 009104) × C57BL/6J (JAX Stock No. 000664) genetic background as a mixed background is most likely to produce the widest range of phenotypes (Doetschman, 2009). The composition of the hybrid background was periodically evaluated using the Jackson Labs Genome Screening Service, and the colony was refreshed either with C57BL/6J or 129S4/SvJaeJ to approximate a 50:50 mix. In all experiments, littermate controls were used.

All animal experiments were performed in accordance with NIH guidelines and were approved by the Children’s Hospital Boston Institutional Animal Care and Use Committee.

### Hippocampal Slice Preparation

**Figure 1:**
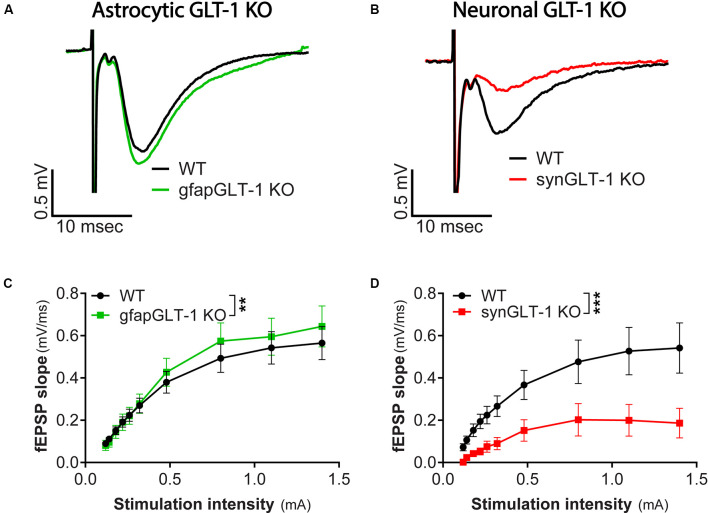
Decreased field potentials in CA1 region of hippocampal slices from synGLT-1 KO but not gfapGLT-1 KO mice. Field excitatory post-synaptic potentials (fEPSPs) were recorded in the stratum radiatum in the CA1 region of hippocampal slices in response to a single electrical stimulus applied to the Schaeffer collateral/commissural fibers in 30–40 weeks old gfapGLT-1 KO (“Astrocytic GLT-1 KO”; **A,C**) and 30–40 weeks old synGLT-1 KO (“Neuronal GLT-1 KO”; **B,D**) mice and wild-type (WT) littermate controls. **(A)** Representative traces from WT (black trace) and gfapGLT-1 KO (green trace) littermates (0.3 mA stimulus). **(B)** Representative traces from WT (black trace) and synGLT-1 KO (red trace) littermates (0.3 mA stimulus). **(C,D)** Input-output relationship of fEPSP slope produced by stimuli between 0 and 1 mA in gfapGLT-1 and WT littermates **(C)** and in synGLT-1 KO and WT littermates **(D)**. ** = *p* < 0.01, *** = *p* < 0.001 in panels **(C,D)** indicate effect of genotype in a linear mixed model.

**Figure 2:**
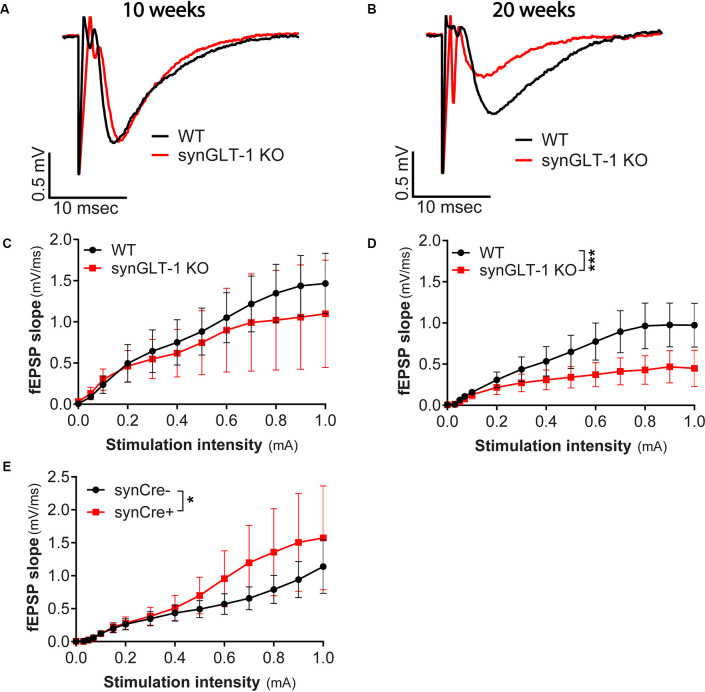
Age-dependent impairment in excitatory synaptic transmission in synGLT-1 KO mice. **(A,B)** Representative fEPSPs recorded in the stratum radiatum of the CA1 region in hippocampal slices in response to a single electrical stimulus applied to the Schaeffer collateral/commissural fibers in 10 weeks **(A)** and 20 weeks old **(B)** WT littermate controls (black traces) and in synGLT-1 KO (red traces). fEPSP slope in 10 weeks old **(C)** and 20 weeks old **(D)** synGLT-1 KO produced by stimuli between 0 and 1 mA. **(E)** Cre-recombinase expression on a true wild-type background (GLT-1^+/+^; 20 weeks) does not impair fEPSP generation. * = *p* < 0.05, *** = *p* < 0.001 in panels **(D,E)** indicate effect of genotype in a linear mixed model.

**Figure 3:**
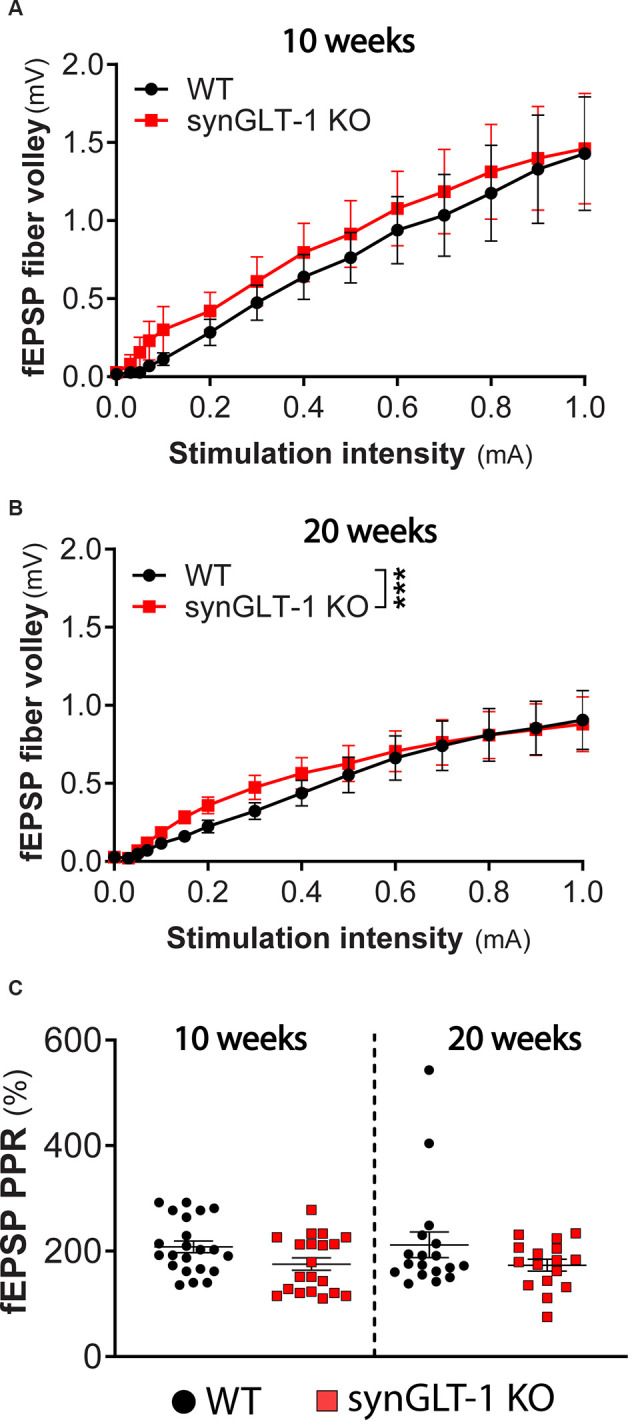
Presynaptic fiber recruitment and paired-pulse facilitation were largely unchanged in synGLT-1 KO compared to wild-type littermates. The fEPSP fiber volley component of field potential recording in the stratum pyramidale of CA1 hippocampal slices in 10 **(A)** and 20 weeks old **(B)** synGLT-1 KO (red traces) and WT littermate controls (black traces) is shown. **(C)** Pair pulse facilitation ratio (PPR) of two paired electrical stimuli at 20 Hz applied to the Schaeffer collateral/commissural fibers in 10 weeks (left) and 20 weeks (right) old WT littermate controls (black symbols) and synGLT-1 KO (red symbols). *** = *p* < 0.001 in panel **(B)** indicates the effect of genotype in a linear mixed model.

**Figure 4:**
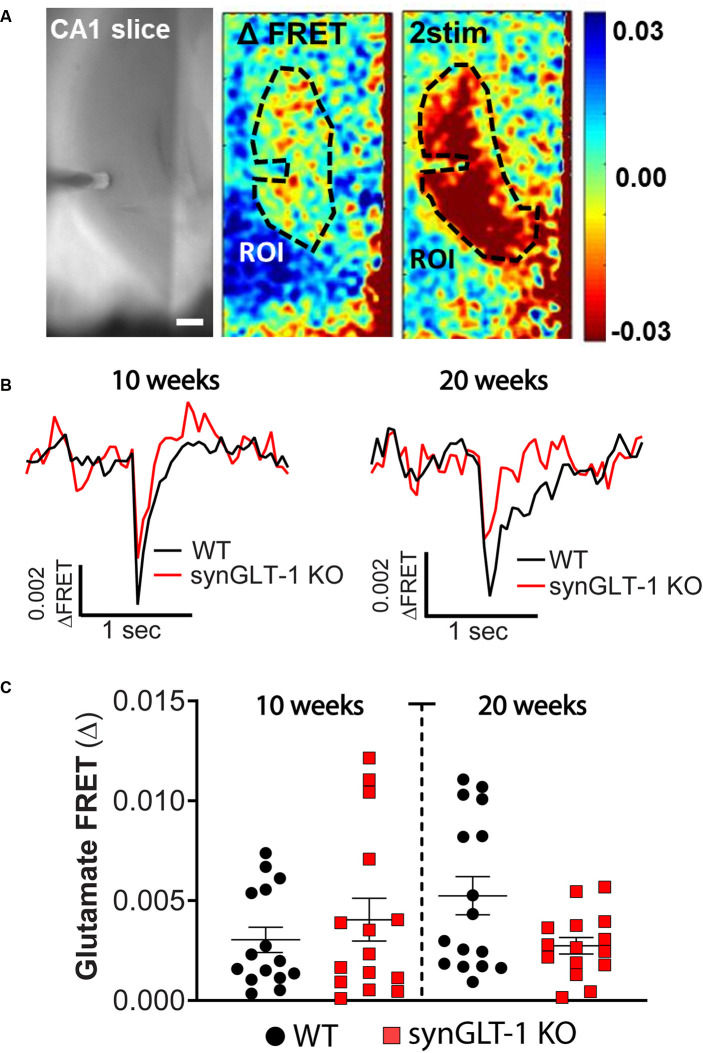
Comparison of stimulus-evoked glutamate release in synGLT-1 KO and wild-type slices. Acute hippocampal slices were prepared from synGLT-1 KO and WT mice and loaded with a glutamate FRET sensor. A recording electrode was placed in the stratum radiatum of CA1 and a bipolar stimulating electrode was placed in the Schaeffer collateral/commissural fibers. **(A)** An example of images from a WT hippocampal slice. Left: phase contrast. Center: pseudo-colored glutamate response after one stimulus (center) and two stimuli at 20 Hz (right) and 0.3 mA intensity. The dashed line in the center and right images indicates the region of interest used in **(B,C)**. **(B)** Single trace of evoked extracellular glutamate FRET biosensor response in 10 weeks (left) and 20 weeks (right) old synGLT-1 KO (red) and WT littermate (black) mice. **(C)** Population of responses obtained at 10 (left) and 20 (right) weeks old synGLT-1 KO and WT mice. The responses in **(B)** and **(C)** were evoked by a single stimulus. There were no significant differences between responses in WT and synGLT-1 KO mice at 10 [Mann-Whitney U 106; *p* = 0.806 *n* = 15 (WT), 15 (KO)] or 20 weeks [(Mann-Whitney U 87; *p* = 0.202 *n* = 15 (WT), 16 (KO)]. Scale bar = 250 micrometers.

**Figure 5:**
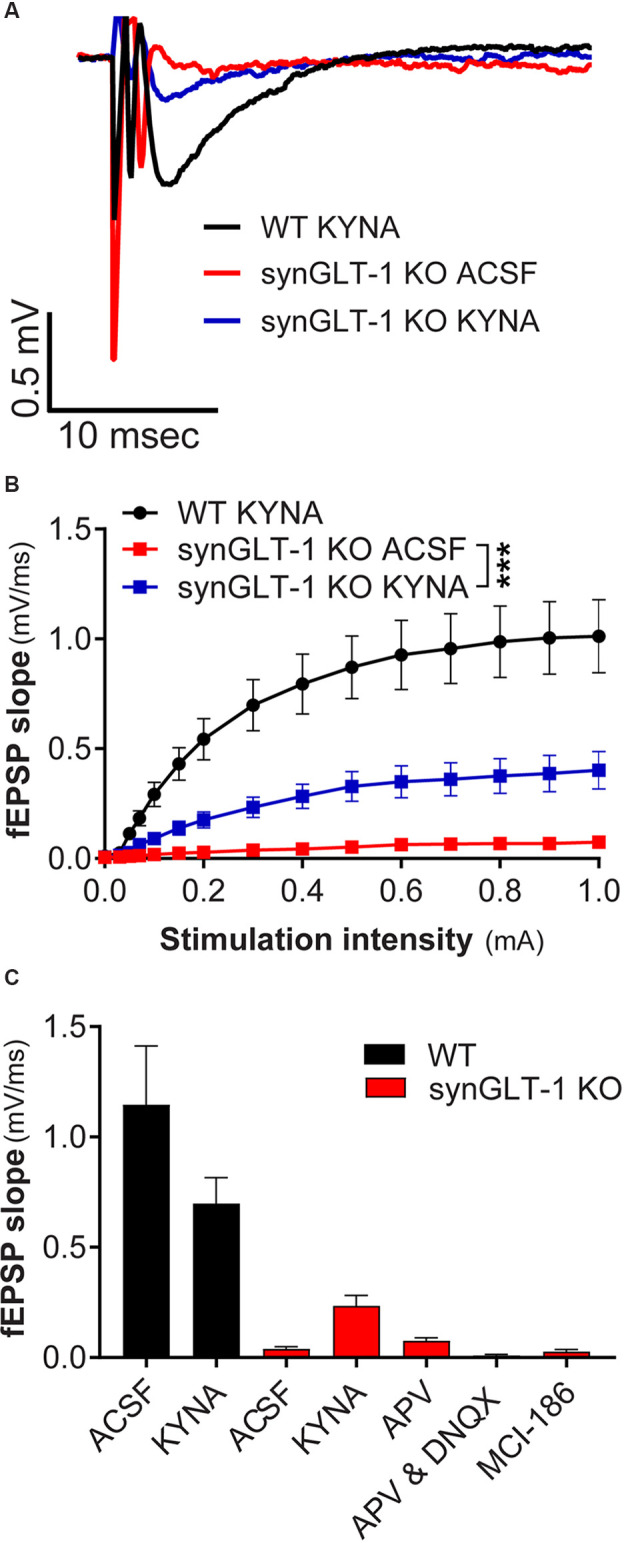
Kynurenic acid partially restored excitatory synaptic transmission in synGLT-1 KO mice. **(A)** Representative field potentials recorded in the stratum radiatum of CA1 hippocampal slices in response to a single electrical stimulus of 0.3 mA applied to the Schaeffer collateral/commissural fibers in 20 weeks old WT littermate controls with 3 mM kynurenic acid treatment (black trace), synGLT-1 KO (red trace) and (synGLT-1 KO with kynurenic acid treatment (blue trace). **(B)** Input-output relationship of fEPSP slope between 0 and 1 mA in 20 weeks old synGLT-1 KO with kynurenic acid treatment (blue squares) or without treatment (red squares) and WT littermates (black circles). **(C)** Summary of data obtained with kynurenic acid and related treatments in ACSF during the recovery period at 0.3 mA stimulus intensity: ACSF, kynurenic acid, 50 μM D-APV, 50 μm D-APV in combination with 20 μM DNQX; MCI-186. *** = *p* < 0.001 in panel **(B)** indicates drug effect in a linear mixed model.

**Figure 6:**
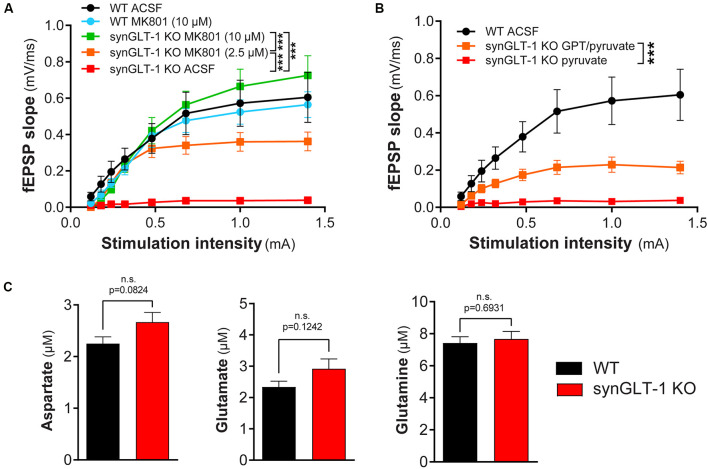
Functional impairment of hippocampal slices was blocked by MK801 or by glutamate-pyruvate transaminase (GPT) in recovery ACSF. **(A)** The non-competitive NMDA receptor blocker MK801 prevented synaptic transmission compromise in synGLT-1 KO slices. Brain slices were allowed to recover in ACSF or ACSF containing MK801 (10 μM or 2.5 μM). Incubation with either 2.5 and 10 μM MK801 significantly increased the fEPSP slope in synGLT1-KO slices. SynGLT-1 KO slices in the presence of 10 μM MK801 (green, 11 slices from four mice) showed a normal input-output relationship compared with WT-ACSF slices (black, nine slices from five mice). A low dose of MK801 (2.5 μM, orange, nine slices from three mice) partially restored fEPSP generation. In ACSF without drug, synGLT-1 KO (red, seven slices from three mice) showed very abnormal I/O. Slices from littermate controls exposed to 10 μM MK801 during the recovery period (WT-MK801, blue, eight slices from two mice) showed responses similar to slices from littermate controls that recovered in ACSF alone (WT-ACSF, black). *** = *p* < 0.001 indicates the effect of drug dose in a linear mixed model. **(B)** The glutamate scavenger system GPT/pyruvate partially prevented synaptic transmission deficits in synGLT-1 KO slices. SynGLT-1 KO slices in the presence of GPT/pyruvate (GPT 10 unit/ml, 10 mM pyruvate, orange, 12 slices from three mice) showed a partially restored input-output relationship compared with slices treated only with pyruvate (red, nine slices from three mice). *** = *p* < 0.001 in panel **(B)** indicates effect of GPT in a linear mixed model. **(C)** Extracellular amino acids following 60 min incubation. Extracellular medium bathing single slices for the recovery incubation period of 60 min was collected and assayed for amino acids. The medium volume was 3 ml. No reliable differences were noted in extracellular glutamate, aspartate, or glutamine concentrations between synGLT-1 KO and wild-type littermate slices.

Mice (synGLT-1 KO and wild-type littermates; gfapGLT-1 KO and wild-type littermates) were euthanized with Isoflurane. The brain was quickly removed and placed in chilled (4°C) low-Ca, high-Mg, low-Na slicing solution consisting of (in mM): 234 sucrose, 11 D-glucose, 24 NaHCO_3_, 2.5 KCl, 1.25 NaH_2_PO_4_, 10 MgSO_4_, and 0.5 CaCl_2_, equilibrated with a mixture of 95% O_2_:5% CO_2_, pH 7.4 (Figures 2–5, 6C). For some experiments, a slightly altered composition for the low-Na slicing solution was used: (in mM) 206 sucrose, 10 D-glucose, 26 NaHCO_3_, 2.8 KCl, 1.0 NaH_2_PO_4_, 2 MgSO_4_, 1 CaCl_2_, 5 MgCl_2_, pH 7.4 (Figures 1A–D, 6A,B). The brain was glued to the slicing stage of a Leica VT1200S Vibratome sectioning system and slices were cut at 350 μm in a coronal orientation. The slices were then incubated in 32°C oxygenated artificial cerebrospinal fluid (ACSF: in mM: 126 NaCl, 2.5 KCl, 1.25 NaH_2_PO_4_, 1 MgSO_4_, 2 CaCl_2_, 10 D-glucose, 26 NaHCO_2_, pH 7.4) for at least 60 min (Figures 2–5, 6C). In some experiments, slices were incubated in ACSF that contained the following (in mM): 124 NaCl, 2.8 KCl, 1.25 NaH_2_PO_4_, 2 MgSO_4_, 2.5 CaCl_2_, 10 D-glucose, 26 NaHCO_3_, 0.4 sodium ascorbate, pH 7.4 (Figures 1A–D, 6A,B). The volume used for the recovery incubation was 300–500 ml (Figures 1A–D, 2A–E, 3, 4) except when drugs were being tested (Figures 5, 6A,B) when the reduced volume (25–100) was used to conserve the drug.

### Electrophysiological Recordings

A single slice was transferred to the recording chamber and continuously perfused with ACSF at ~34°C that had been saturated with 95% O_2_ and 5% CO_2_ after recovery at the same temperature. In some experiments, recording (and recovery incubation) was performed at 26°C (Figures 1A–D, 6A,B). For field recording and glutamate imaging, this was done in an interface chamber; in some experiments, submerged slices were used (Figures 1A–D, 6A,B). Whole cell recording was performed using submerged slices.

A bipolar stimulating electrode (FHC Inc., Bowdoin, ME) was placed in the Schaeffer collaterals to deliver stimuli. A borosilicate glass recording electrode filled with ACSF was positioned in the stratum radiatum of CA1, 200–300 μm from the stimulating electrode. The input/output relationship was determined with a series of increasing stimulation intensities. Paired pulse facilitation of fEPSP was tested by two stimuli 50 ms apart with an intensity of 300 μA. Electrophysiological data were recorded with an Axon Multiclamp 700A amplifier or with an Axon Instruments 200 B amplifier and Digidata 1322A digitizer (sampling rate = 10 or 20 kHz; filtered at 2 kHz) with pClamp software (Molecular Devices).

Recovery times were similar between genotypes, and the minimum time for recovery was 60 min. Slices were taken as needed from the recovery ACSF for recording after the 60-min recovery period. Although the first slices were recorded following 60 min recovery period, the last to be recorded on a given day may have been in the recovery ACSF for up to 4 h. No effect was observed of time in recovery ACSF on the phenotype observed in the synGLT-1 KO. In all cases comparing wild-type and KO slices, slices from wild-type and KO littermates were compared on the same experimental day. Therefore, slices from both mutant and control genotypes were always subjected to the same experimental conditions.

### Glutamate Biosensor Imaging

Production, loading of glutamate biosensor, and collection of biosensor data were performed as previously described (Dulla et al., 2008). A 35 mm tissue culture dish was filled with ~2 ml ACSF and a 0.4 μm Millicell (Millipore) culture plate was inserted. Care was taken to ensure that no bubbles were present under the plate insert and that no ACSF spilled onto its top surface. A single brain slice was transferred from the incubation chamber onto the plate insert and excess ACSF was removed. The dish containing the slice was then placed in a humidified and warmed (32°C) chamber equilibrated with 95% O_2_:5% CO_2_. Fifty microliter of concentrated glutamate FRET sensor protein (≈ 50 ng/μl) was then carefully applied to the top surface of the slice. After 5–10 min of incubation, slices were removed from the loading chamber and placed into the recording chamber.

Slices were placed into the recording chamber of an Olympus Bx51WI microscope with continual superfusion of ACSF for simultaneous imaging with an Olympus 4× objective. Excitation with 440 nm wavelength light was used. Imaging was performed using a Zyla (Andor) camera imaging at 200 Hz, illuminated by a 480 nm LED (Thorlabs), using the Endow-GFP filter cube (Chroma) and controlled by MicroManager (Edelstein et al., 2014). Each imaging experiment consisted of collecting 500 images at 5 ms acquisition time. A direct trigger sent from the camera triggered evoked stimuli delivery by stimulation isolator after the acquisition of 20 images. Emission signals first passed through a 455 nm DCLP dichroic mirror to eliminate excitation fluorescence and were then separated into two channels using a Photometrics Dual-View or Optosplit two channel imaging system to isolate cyan fluorescent protein (CFP) and Venus, a variant of yellow fluorescent protein (YFP), signals.

Regions of interest (ROIs) were manually drawn within the CA1 stratum radiatum symmetrically around the stimulation electrode to include regions from both the CA1 and CA3 sides of the electrode. Raw imaging data was first split into CFP and Venus and the ratio of the two fluorophores was computed. An average pre-stimulation ratio image was then made by averaging the first three images. The pre-stimulation image was then subtracted from all images resulting in a ΔFRET image. Processed ΔFRET images were then converted into ΔFRETsignal/ΔFRETnoise data, pixel-by-pixel, by dividing all time-points by the standard deviation of ΔFRET during the pre-stimulus time period. Bleaching of the biosensor was corrected by calculating an exponential function fit. Imaging data were then analyzed to determine the peak amplitude of the signal.

### Assay of Extracellular Amino Acids

#### Extracellular Amino Acids

Medium (*V* = 3 ml) incubating single slices during the recovery period of 60 min was collected and assayed for amino acids. Slices from a single animal were used for each experiment and wild-type and synGLT-1 KO animals were used on sequential days. The medium was kept frozen at −20°C after collection and until assay. At the time of assay, 0.9 ml medium was added to 0.1 ml 35% sulfosalicylic acid to remove proteins. The acidified medium was kept on ice for 20 min, after which it was centrifuged, and the supernatant was decanted and assayed. Amino acid content was determined by the Biochemical Genetics Laboratory at the Kennedy Krieger Institute using a Biochrom 30 amino acid analyzer (Pei et al., 2010). In these experiments, slices from single animals were incubated for selected times in ACSF, incubated at 34°C. Wild-type and KO animals were run on sequential days. Three pairs were assayed, four slices per animal on a given experimental day, for a total of 12 slices/genotype.

### Glutamate Uptake and Metabolism in Hippocampal Brain Slices

Glutamate uptake and metabolism were investigated by incubation of acutely isolated hippocampal mouse brain slices as previously described (Andersen et al., 2021b). The mouse was euthanized by cervical dislocation and the brain transferred to ice-cold artificial cerebrospinal fluid (ACSF) containing in mM: 128 NaCl, 25 NaHCO_3_, 10 D-glucose, 3 KCl, 2 CaCl_2_, 1.2 MgSO_4_, 0.4 KH_2_PO_4_, pH 7.4. The hippocampi were dissected and sliced (350 μm) using a McIlwain tissue chopper (The Vibratome Company, O’Fallon, MO, USA). The hippocampal slices were kept just below the surface of 10 ml 37°C oxygenated (5% CO_2_/95% O_2_) ACSF and pre-incubated for 60 min. Subsequently, the media were exchanged for ACSF (with an adjusted D-glucose concentration of 5 mM) containing 200 μM [U-^13^C]glutamate and incubated for additional 60 min. Incubations were terminated by transferring the slices into ice-cold 70% ethanol. Slices were subsequently sonicated and centrifuged (4,000 *g* × 20 min) and the supernatant was removed and lyophilized before further analysis. The protein content of the pellets was determined by Pierce protein assay. The ^13^C enrichment of tricarboxylic acid (TCA) cycle intermediates and amino acids from [U-^13^C]glutamate metabolism was determined by gas chromatography-mass spectrometry (GC-MS) analysis. Slice extracts were reconstituted in water, acidified, extracted with ethanol and the metabolites were derivatized using *N*-tert-butyldimethylsilyl-*N*-methyltrifluoroacetamide. Samples were analyzed by GC (Agilent Technologies, 7820A, J&W GC column HP-5 MS) coupled to MS (Agilent Technologies, 5977E). The isotopic enrichment was corrected for the natural abundance of ^13^C by analyzing the standards of the unlabeled metabolites of interest. The expected labeling pattern of [U-^13^C]glutamate metabolism is described in Andersen et al. (2017a) and data is presented as M + X, where M is the molecular ion and X is the number of ^13^C atoms in the molecule. Aqueous slice extracts were further analyzed by reverse-phase high-performance liquid chromatography (HPLC, Agilent Technologies, 1260 Infinity, Agilent ZORBAX Eclipse Plus C18 column) to quantitatively determine the amounts of amino acids (Andersen et al., 2017b). Ten male mice of each genotype, 20 mice in total, were used for the metabolic slice experiments.

One mouse was used at a time for experiments. For each incubation (20 in total, 10 WT and 10 KO), 5–6 hippocampal slices were tested/condition i.e., incubated together exposed to [U-^13^C]glutamate. All of the 5–6 slices were then homogenized and analyzed together to provide one data point.

### Electron Microscopic Immunocytochemical (EM-ICC) Detection of GLT-1 and Analysis of Hippocampal Mitochondria

The procedures used were exactly as reported previously (Petr et al., 2015) and are described only briefly here. Animals, all male, were transcardially perfused under anesthesia at 22–24 weeks of age, in one session, using a mixture of 0.1% glutaraldehyde/4% paraformaldehyde in 0.1 M phosphate buffer (PB, pH 7.4) to fix their brains. All subsequent steps for tissue preparation were conducted strictly in parallel, so as to minimize inter-animal differences in ultrastructural preservation due to unintended differences in tissue handling or of chemical reagents.

Brains were cut at a coronal plane using a vibrating microtome, with thickness of sections set to 50 μm. Brain sections spanning the dorsal hippocampus were collected, treated with sodium borohydride (1%, in PB, pH 7.4) for 30 min, then rinsed in PB and stored for a month in a 4°C cold room, free-floating in PBS (phosphate buffered saline, pH 7.6) that contained 0.05% sodium azide. EM-ICC was achieved by using a monoclonal anti-GLT-1a antibody at a dilution of 1:10,000 (generous gift of Dr. Jeff Rothstein, Johns Hopkins U) and detected using HRP-DAB/osmium.

Ultrastructural analysis was performed strictly from the stratum radiatum of CA1 of the dorsal hippocampus, after capturing electron microscopic images at a magnification of 40,000× from portions of the vibratome section that were most superficial and thus optimal for immunodetection. Synaptic neuropil that fulfilled these two criteria were captured systematically, strictly in the order of encounter, while kept blind to the genotype of the animal. Encounter with asymmetric (presumably excitatory) synapses was recorded, together with the presence vs. absence of GLT-1 immunoreactivity and presence vs. absence of mitochondria within the presynaptic axon terminal. The rate of encounter with GLT-1 immunoreactivity was measured for every group of 10 excitatory synapses, and this assessment of the rate of the encounter was repeated approximately 20 times, thereby assessing the rate of encounter of a neuropil region spanning 200 or more excitatory synapses. Within the mitochondria encountered, the average distances between cristae were calculated as described below, under “Statistical Analyses”.

### Statistical Analyses

#### Electrophysiology and Biochemistry

For comparison between two experimental groups other than electron microscopy, Student’s unpaired t-test was used. Values of *p* < 0.05 or less were considered statistically significant. For cases in which the same brain slice was stimulated multiple times, and other repeated measures, linear mixed modeling (LMM) was used to examine the effects of genotype or drug treatment. This approach estimates the effect size of each factor while accounting for intra- and inter-animal variability (Aarts et al., 2014; Boisgontier and Cheval, 2016; Yu et al., 2021) and is gaining wide acceptance (Lau et al., 2017; Huang et al., 2018; Hanson et al., 2019; Koenig et al., 2019; Grieco et al., 2020; Kurucu et al., 2021). LMMs were fitted with random intercepts to assess for the correlation between repeated measurements on the same mouse, and experiment-specific effects were analyzed for statistical significance. LMM was performed in R-Studio using the lme4 and lmreTest libraries. Slices were considered nested within animals when considering intra- vs. inter-animal variability. For each test examining the effect of genotype, R code similar to the following was used “output ~ (Amp + Amp: Genotype) + (1 | Animal) + (1 | Animal: Slice)” where “output” is the measure of interest, “Amp” is the stimulation intensity and “genotype” is the animal genotype. For each test examining the effect of drug treatment, an R code similar to the following was used “output” ~ (Amp * Genotype * drug) + +(1 | Animal) + (1 | Animal:Slice) where “drug” was the dose of drug used. For no drug controls, “drug” equaled zero, values >1.96 and < −1.96 were considered to be statistically significant and corresponded to 95% confidence intervals that did not cross zero. Each LMM examined both main fixed effects (genotype, drug treatment, stimulation intensity, etc.) and interactions between the effects.

#### Electron Microscopy

GLT-1 immunoreactivity revealed the genotype of the animal to be wild-type or synGLT-1 KO. After this identification, and genotype validation from tail DNA that was collected prior to euthanasia, the values of the rate of encounter with GLT-1 immunoreactive presynaptic terminals were pooled across animals of the same genotype. The number of pooled values was equalized across animals (~20). Normality test indicated failure by the Anderson-Darling, D’Agostino and Pearson, Shapiro-Wilke, and Kolmogorov-Smirnov tests. Thus, the Mann-Whitney test was used to determine the significance of the difference between the median values from wild-type vs. synGLT-1 KO tissue. Graphpad Prism (version 9 for MacOS) was used to perform these tests and to plot the graphs.

The frequency of encounters with mitochondria within presynaptic terminals of excitatory synapses was assessed as described above for assessing the frequency of encounters with GLT-1 immunoreactivity.

#### Mitochondrial Cristae Density

Mitochondria occurred in both GLT-1 labeled and unlabeled axon terminals forming excitatory synapses. The average distance between cristae was calculated to assess cristae density. The distance, d, spanning from one crista to another or two neighboring cristae within a single mitochondrion was measured, using ImageJ’s tool for measuring the lengths of line segments (version 2.1.0/1.53c). The average distance between neighboring cristae of a mitochondrion was calculated based on the following formula: Average distance = d/(number of cristae-1). The average distance values of mitochondria were pooled across animals of the same genotype (~30 mitochondria per animal), then compared across genotype groups using the Mann-Whitney test, as described above. The pooled samples were equalized in sample size across animals.

Error bars used in the data presentation in the figures represent the standard error of the mean (SEM) throughout.

### Drugs and Reagents

All salts and glucose for buffers and other reagents were obtained from Sigma-Aldrich, except as noted. MCI-186 was obtained from Cayman Chemical. MK801 was obtained from Tocris. D-APV was from Tocris and Abcam. DNQX was from Tocris.

## Results

### Hippocampal Field Excitatory Postsynaptic Potentials in Neuron- and Astrocyte- Specific GLT-1 KO

We first recorded field excitatory postsynaptic potentials (fEPSPs) in the CA1 region of gfapGLT-1 KO and synGLT-1 KO mice, and compared responses with wild-type littermate controls (WT). Schaeffer collaterals were stimulated and fEPSPs from pyramidal neurons in the CA1 region of the hippocampus of 30–40 weeks old gfapGLT-1 KO (Figure 1A) and WT mice were recorded. fEPSP slope [Figure 1C; LMM, *t* = 3.15, *p* = 0.0017, interaction of stimulation intensity and genotype, *n* = 18 slices from seven animals (WT), 18 slices from five animals (KO)] was slightly, but significantly increased, compared to WT mice. We next measured field responses in 25–40 weeks old synGLT-1 KO (Figure 1B) and WT mice and found that the fEPSP slope [Figure 1D; LMM, *t* = 5.393, *p* = 2.28e-07, *n* = 10 slices from four animals (WT), nine slices from seven animals (KO)] of the neuronal GLT-1 KO was significantly decreased compared to WT mice. As such, the loss of GLT-1 from presynaptic terminals but not from astrocytes appeared to negatively affect synaptic transmission in the CA1 region of hippocampal slices.

### Age-Dependent Decrease of fEPSP in the Neuronal GLT-1 KO

Since we observed abnormal field responses in the synGLT-1 KO but not gfapGLT-1 KO mice, we next focused on characterizing this defect in the synGLT-1 KO further. We recorded fEPSPs of synGLT-1 KO and WT mice at 10 (Figures 2A,C) and 20 weeks of age (Figures 2B,D). At 10 weeks of age, we found that fEPSP slope [LMM, interaction of stimulation and genotype, *t* = −1.93, *p* = 0.053; *n* = 15 slices from five animals (WT), *n* = 15 slices from four animals (KO)] was not significantly decreased in synGLT-1 KO, as compared to WT mice (Figures 2A,C). In contrast, at 20 weeks, fEPSP slope (*t* = −5.34, *p* = 1.45e-7) was significantly reduced (53% reduction) in the synGLT-1 KO compared with the WT [*n* = 18 slices from six animals (WT), 17 slices from five animals (KO); Figures 2B,D]. These results suggest an age-dependent defect in fEPSP generation in synGLT-1 KO hippocampal slices.

### The Expression of Cre Does Not Decrease fEPSPs

To exclude the possibility that the decrease in fEPSPs in synGLT-1 KO that we observed was due to expression of Cre-recombinase, *per se*, we used synapsin-Cre control mice, i.e., animals that express Cre, but on a WT genetic background, and their WT littermates (synCre+ and synCre-). Expression of Cre caused a small, but significant, increase in fEPSP slope [Figure 2E; LMM, interaction of stimulation and genotype, *t* = 2.48, *p* = 0.02; *n* = 10 slices from three animals (WT), *n* = 11 slices from four animals (KO)] in slices from animals at 20 weeks of age. These data indicate that the decrease in fEPSPs in synGLT-1 KO is not the result of Cre-recombinase expression, but rather, from a loss of GLT-1 from the presynaptic terminal.

### Presynaptic Fiber Recruitment of Schaeffer Collaterals and Paired Pulse Facilitation Are Largely Unaffected in synGLT-1 KO

The fiber volley reflects the excitation of axons projecting into the dendritic field recording site and produces a small peak that occurs prior to the fEPSP (Otmakhova and Lisman, 1999; Kim et al., 2012; Tani et al., 2014). The amplitude of this phenomenon did not significantly differ in 10 weeks old synGLT-1 KO mice, as compared to WT [Figure 3A; LMM, interaction of stimulation and genotype, *t* = -0.12, *p* = 0.91, *n* = 15 slices from five animals (WT), *n* = 15 slices from four animals (KO)]. At 20 weeks of age, the fiber volley was slightly but significantly increased in synGLT1-KO mice compared to WT [Figure 3B; LMM, interaction of stimulation and genotype, *t* = 6.865 *p* = 7.15e-11, *n* = 18 slices from six animals (WT), *n* = 17 slices from five animals (KO)], indicating that the presynaptic fiber recruitment of Schaeffer collaterals is, at most, mildly affected by the loss of presynaptic neuronal GLT-1. Paired pulse ratio (PPR) is an important measure of presynaptic function, sensitively reflecting changes in calcium dynamics in the presynaptic terminal (Schulz et al., 1994, 1995; Wu and Saggau, 1994; Mukhamedyarov et al., 2006; Krall et al., 2020), and may be influenced by fast inhibitory transmission as well (Nathan et al., 1990; Nathan and Lambert, 1991; Stuart and Redman, 1991). We found no significant difference in PPR in 10 weeks [*p* = 0.0928, *n* = 22 slices from six animals (WT), *n* = 17 slices from five animals (KO)] and 20 weeks old synGLT-1 KO mice compared to WT [*p* = 0.6455, *n* = 18 slices from six animals (WT), *n* = 16 slices from five animals (KO); Figure 3C]. The lack of a change in PPR suggests that the impairment of fEPSP generation observed in the synGLT-1 KO hippocampal slices is not due to a compromise of presynaptic function, for example, by impaired calcium dynamics (Wu and Saggau, 1994; Dittman et al., 2000; Mukhamedyarov et al., 2006).

### Glutamate Imaging in Hippocampal Slices of Neuronal GLT-1 KO

Expression of GLT-1 in axon terminals might be required to maintain stores of the neurotransmitter glutamate, and also might participate in the clearance of glutamate from the extracellular space after release. We tested whether stimulus-evoked extracellular glutamate accumulation was affected by neuronal GLT-1 KO by measuring stimulus-evoked changes in extracellular glutamate using a FRET-based glutamate sensor (Figure 4). These glutamate imaging studies revealed that evoked glutamate release was only marginally, but not significantly, reduced in 20 weeks old synGLT-1 KO compared to WT mice [Mann-Whitney U 87; *p* = 0.202 *n* = 17 slices from five animals (WT), *n* = 16 slices from (KO); Figure 4C], and there was no difference in evoked glutamate release in synGLT-1 KO mice compared to WT mice at 10 weeks [Mann-Whitney U 106; *p* = 0.806 *n* = 15 slices from five animals (WT), *n* = 15 slices from four animals (KO)]. These results provide additional evidence that the impairment of fEPSP generation in the synGLT-1 KO slices is not due to a presynaptic deficit of neurotransmitter glutamate, and also argue against a significant role for neuronal GLT-1 in glutamate clearance.

### MK801 in the Recovery ACSF Restores fEPSP Generation

We intended to use patch clamp recordings to further pursue the cellular basis of the age-dependent deficit in synaptic transmission observed in the synGLT-1 KO. Upon optical assessment after slice preparation and a 1 h resting period, pyramidal cells in the stratum radiatum of the CA1 region in acute brain slices of 20 weeks old synGLT-1 KO were found to be significantly swollen compared to WT littermates, and it was not possible to obtain stable patch-clamp recordings from them. Impaired glutamate uptake can lead to excessive activation of excitatory amino acid receptors and cell death, a process known as excitotoxicity (Rothman and Olney, 1986; Choi, 1988; Meldrum and Garthwaite, 1990; Lipton and Rosenberg, 1994). Excitotoxicity produces an initial phase of cell swelling of neurons that precedes cell death (Choi, 1985; Choi et al., 1987; Ramnath et al., 1992; Churchwell et al., 1996). Kynurenic acid is an antagonist of all ionotropic glutamate receptors and is known to prevent excitotoxicity (Ganong et al., 1983; Foster et al., 1984; Espanol et al., 1994; Pozzo Miller et al., 1994; Urenjak and Obrenovitch, 2000; Feher et al., 2019; Toth et al., 2021). We added kynurenic acid (3 mM) to the recovery ACSF after slicing to attempt to prevent possible excitotoxicity in the synGLT-1 KO acute hippocampal slices. We then compared fEPSPs of 20 weeks old synGLT-1 KO and WT mice with or without kynurenic acid treatment (Figures 5A–C). In synGLT-1 KO animals kynurenic acid significantly increased fEPSP slope from 0.039 ± 0.0106 mV/ms (synGLT1 ACSF) to 0.235 ± 0.047 mV/ms (synGLT1 KYNA) [LMM, interaction of stimulation and dose effect, *t* = 12.20, *p* = 2e-16; *n* = 16 slices from four animals (KO ACSF), *n* = 16 slices from four animals (KO KYNA)], *n* = 15 slices from four animals (WT KYNA; Figure 5B) representing 34% of the WT KYNA fEPSP slope (0.699 ± 0.117mV/ms) at 0.3 mA stimulation. The partial recovery of function produced by kynurenic acid in the recovery ACSF was consistent with the possibility that excitotoxic injury might be occurring in the synGLT-1 KO slices.

To ascertain whether the effect of kynurenic acid was due to blocking glutamate receptors we tested the effects of the competitive NMDA receptor antagonist 2-amino-5-phosphonovalerate (D-APV; 50 μM; Davies et al., 1981; Kass et al., 1989) alone or in combination with DNQX (20 μM), which is a competitive inhibitor of non-NMDA receptors (Honore et al., 1988; Sheardown et al., 1990). Surprisingly, APV, alone [Mann-Whitney U = 8, *p* = 0.0719, *n* = 17, 3; 0.3 mA; *n* = 3 slices from one animal (KO plus APV)] or in combination with DNQX [Mann-Whitney U = 29, *p* = 0.0553, *n* = 17, 7; 0.3 mA; *n* = 7 slices from two animals (KO plus APV/DNQX, *n* = 17 slices from four animals (KO no drugs)], did not produce a significant recovery of the fEPSP slope in synGLT-1 KO mice (Figure 5C). Kynurenic acid itself did not have a significant effect on the fEPSP in slices from WT animals [*n* = 14 slices from five animals WT), *n* = 16 slices from five animals (WT plus KYNA)]. Since kynurenic acid was used here at 3 mM, well above its affinity for glutamate receptors (Albuquerque and Schwarcz, 2013), we considered the possibility that other actions of kynurenic acid might be involved, for example, oxygen-free radical scavenging properties (Lugo-Huitron et al., 2011; Gonzalez Esquivel et al., 2017). Therefore, we tested a membrane-permeable reactive oxygen species (ROS) scavenger (MCI-186, 33 μM; Wu et al., 2006; Schurr and Gozal, 2012). Addition of this compound to the recovery ACSF (Mann-Whitney U = 23, *p* = 0.8421, *n* = 17, 3; 0.3 mA; *n* = 3 slices from one animal) did not improve neurotransmission in synGLT-1 (Figure 5C). We found that kynurenic acid at 500 μM had no effect (data not shown), making it unlikely that it could be acting at nicotinic receptors, to which kynurenic acid binds with high affinity (Albuquerque and Schwarcz, 2013).

Since excitotoxicity in acute slices is primarily due to excessive activation of NMDA receptors (Feig and Lipton, 1990), we tested MK801, a non-competitive NMDA receptor antagonist. We found that 10 μM MK801 in the recovery medium promoted full recovery of fEPSP generation (WT-ACSF, *n* = 9 slices from five animals, WT-MK801, *n* = 8 slices from two animals, synGLT-1 KO MK801 10 μM, *n* = 11 slices from four animals; Figure 6A), and 2.5 μM was also effective (synGLT-1 KO MK801 2.5 μM, 9 slices from three animals), producing 65% recovery of the fEPSP slope [LMM, interaction of stimulation and dose effect, *t* = −10.43, *p* = 2e-16 compared with WT MK801]. These data suggested that excitotoxic injury prevented functional recovery of acute slices derived from the synGLT-1 KO. Excitotoxicity might be due to excess accumulation of excitatory amino acids in the extracellular medium or increased sensitivity to normal extracellular glutamate concentrations due to metabolic compromise (Novelli et al., 1988; Henneberry et al., 1989a, b). To test the possibility that excitotoxic injury in the synGLT-1 slices might be due to extracellular glutamate, we used a glutamate scavenging system (O’Brien and Fischbach, 1986; Blitzblau et al., 1996) to remove glutamate from the extracellular medium (Figure 6B). We found that glutamate pyruvate transaminase (GPT) in combination with pyruvate added to the recovery medium provided partial protection and recovery of fEPSP generation [34%; LMM, interaction of stimulation and drug, *t* = −5.66, *p* = 7.86e-8; *n* = 9 slices from three animals (KO pyruvate); *n* = 12 slices from three animals (KO GPT/pyruvate); Figure 6B]. Pyruvate alone had no effect (Figure 6B), nor did α-ketoglutarate, one of the products of the reaction driven by GPT (data not shown). We assayed excitatory amino acids in the medium bathing the slices during the recovery period. Both glutamate and aspartate were detectable, but there was no significant difference in either in the media bathing the synGLT-1 KO and WT control slices following a 60-min incubation (Figure 6C; pooled data from three separate experiments for each genotype, medium incubated with each of four slices assayed individually/genotype/experiment × 3 experiments = 12 slices/genotype; 30–47 weeks old animals), consistent with the normal expression of astrocytic GLT-1 in both the synGLT-1 KO and WT control slices.

### Ultrastructural Changes in Mitochondria in the CA1 Region in synGLT-1 KO Mice

**Figure 7:**
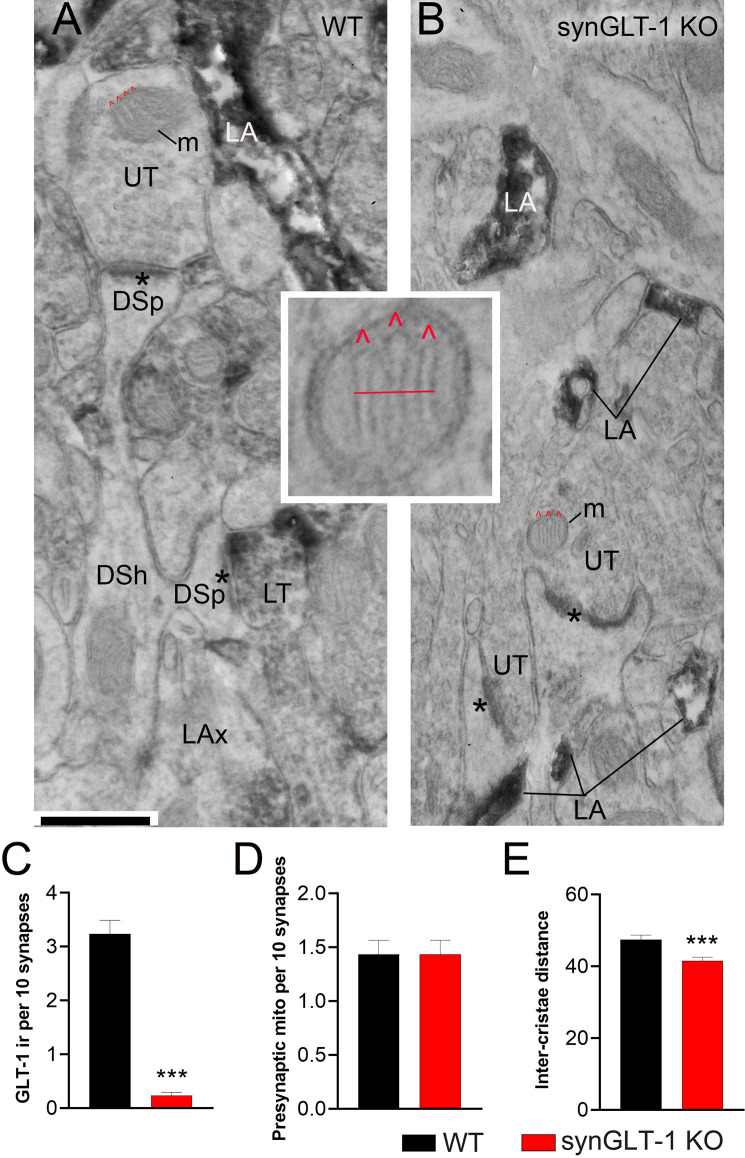
Neuronal GLT-1 knockout decreased inter-cristae distance of presynaptic mitochondria. Panels **(A,B)** show examples of electron micrographs taken from the WT **(A)** vs. synGLT-1 KO **(B)** hippocampus at 22–24 weeks of age. Within the synaptic neuropil of WT animals, only a fraction of axon terminals forming asymmetric synapses with thick PSDs (asterisks; presumably excitatory) upon dendritic spines (DSp) are GLT-1 immunolabeled (LT, abbreviation for “labeled terminal”). Immunolabeling within the axon terminal is evident, based on the diffuse distribution of electron-dense material reflecting the HRP-DAB reaction product. Immunolabeling extends beyond the terminal portion of axons (LAx, abbreviation for “labeled axons”). Axon terminals lacking immunolabeling are labeled as UT (unlabeled terminals). As expected, LT is scarce within KO tissue but both synGLT-1 KO and WT tissue exhibit intense GLT-1 immunolabeling of astrocytic processes (LA, abbreviation for “labeled astrocyte”). DSh = dendritic shaft. Each panel shows examples of mitochondria (m) within axon terminals, with cristae indicated using the symbol ^∧^ in red. The small panel in the center highlights the presynaptic mitochondrion from panel **(B)**, magnified 4x beyond the magnification *via* the electron microscope, to depict the distance (red line) measured spanning the three cristae. Inter-crista distance was calculated as this distance, divided by the number of cristae minus 1 (3–1 = 2 for this example). Calibration bar = 500 nm. Graphs **(C–E)** show group mean averages of GLT-1 immunoreactivity **(C)**, frequency of occurrence of mitochondria within presynaptic axon terminals forming excitatory synapses **(D)**, and average inter-crista distance of mitochondria within presynaptic axon terminals. For all graph panels, the repeated measure of occurrence of the ultrastructural element per every 10 synapses was pooled across three animals of the same genotype (for **C**, *n* = 67 repeated measures for 670 synapses within WT tissue and *n* = 60 repeated measures for 600 synapses within synGLT-1 KO tissue; for **D**, *n* = 60 repeated measures for 600 synapses each from WT and synGLT-1 KO tissue). For **(C)**, the Mann Whitney t-test revealed significant genotype difference (*p* < 0.0001, Mann Whitney U 168.5). For **(E)**, the inter-crista distance values were pooled across animals of the same genotype (*n* = 87 mitochondria for WT, *n* = 90 mitochondria for synGLT-1 KO). Mann-Whitney test revealed significant genotype difference (*p* = 0.0008, Mann Whitney U 2784) *** = *p* < 0.001.

We have previously reported that synGLT-1 KO mice at 8–10 weeks of age had increased density of mitochondria in synaptic terminals in the cortex and hippocampus, and increased cristae packing density in these two regions as well as in the striatum, possibly an adaptive response to decreased access to glutamate as a substrate for synaptic mitochondrial metabolism (McNair et al., 2019, 2020). Since the electrophysiological phenotype we observed occurred in slices from 20 weeks old mice, we wanted to determine whether similar ultrastructural changes were present in this older cohort of mice (Figure 7).

Electron microscopic analysis verified the genotype of the animals to be WT vs. synGLT-1 KO (Figures 7A–C). Astrocytic immunocytochemical labeling for GLT-1a was intense for both genotypes within WT and synGLT-1 tissue of the stratum radiatum of dorsal CA1 of the hippocampus (Figures 7A,B). The HRP-DAB reaction product was associated with the astrocytic plasma membrane and had diffused intracellularly, but leaving the lumen of intracellular organelles, such as vesicles and mitochondria unlabeled. In comparison, GLT-1 immunoreactivity within axon terminals was less intense but still identifiable, and distinctly more electron-dense than the neighboring mitochondria. Most axon terminals in the vicinity exhibited equal electron density across pre- and post-synaptic sides and no greater electron density than mitochondria or postsynaptic densities (PSDs). Such axon terminals were categorized as unlabeled (UL). Using these criteria to judge immunoreactivity in a blinded analysis, the frequency of encounter with GLT-1 immunoreactive axon terminals forming excitatory synapses was significantly higher for tissue from WT animals (median value of 3 per 10 synapses) than for tissue from synGLT-1 KO animals (median value of 0 per 10 synapses) [*p* < 0.0001, Mann-Whitney U 168.5; *n* = 3 animals per genotype, *n* = 67 sections (WT), *n* = 60 sections (KO)]. As further validation of the genotype, tissue of the WT animals, but not of the synGLT-1 KO animals exhibited axons of passage, without synaptic contacts but with GLT-1 immunoreactivity.

The same electron micrograph sets were used to assess the frequency of mitochondria within axon terminals forming excitatory synapses (Figure 7D). This analysis revealed a remarkable similarity across the genotypes (*p* > 0.9999, the median value of 1.500 per 10 synapses for both genotypes; Mann-Whitney U 1800; *n* = 60 for both genotypes). This result indicates that at 20 weeks of age altered excitatory synaptic transmission within the hippocampus of synGLT-1 KO brains did not perturb the size or rate of autophagy of mitochondria (Eskelinen et al., 2011) to culminate in altered mitochondrial presence within synaptic terminals forming excitatory synapses.

The same electron micrograph sets were used to assess the average distances between cristae of mitochondria within axon terminals forming excitatory synapses (Figure 7E). This analysis revealed a significant difference in the average distance between neighboring cristae within single mitochondria. The average distance was lower for the synGLT-1 KO tissue (median 40.42 nm, *n* = 90 mitochondria), compared to WT (46.25 nm, *n* = 87 mitochondria). This 17% difference was statistically significant (*p* = 0.0008, Mann-Whitney U 2784). These data show that the changes in cristae density previously reported in 8–10 weeks old mice persist in 20–25 weeks old mice.

### Impaired Glutamate Metabolism in Hippocampal Slices of Neuronal GLT-1 KO

**Figure 8:**
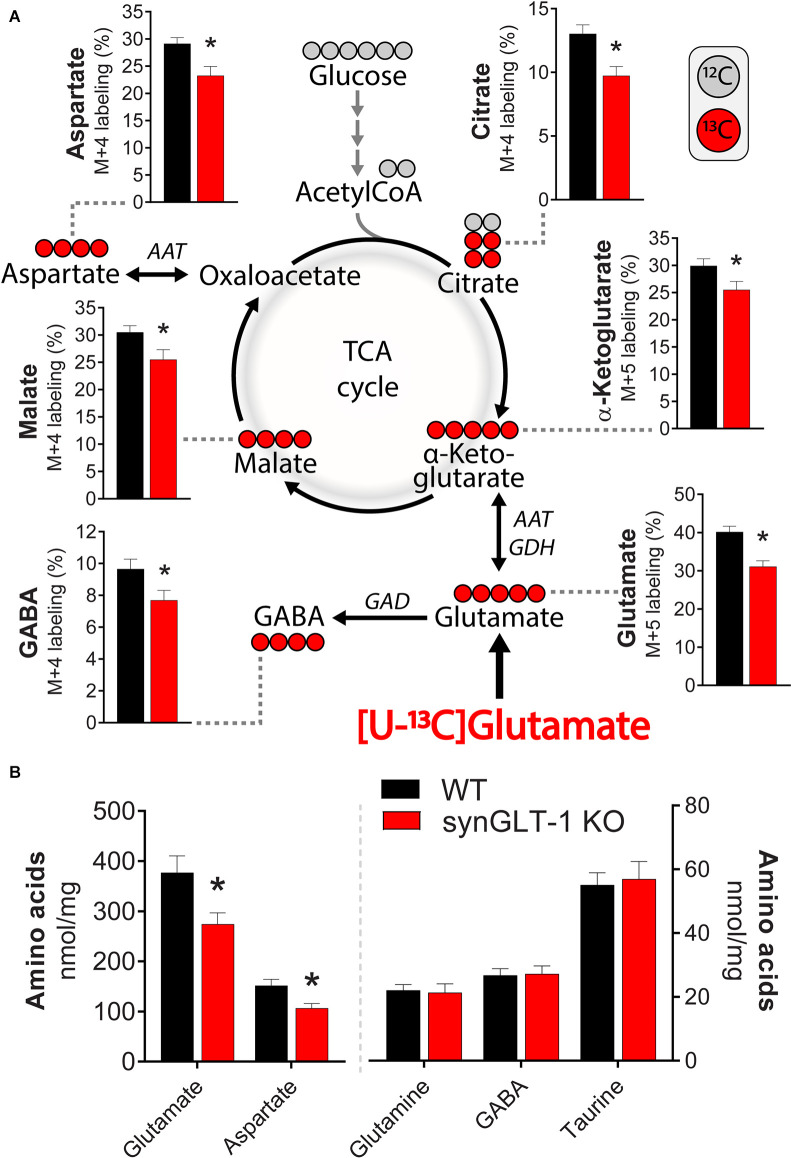
Deletion of neuronal GLT-1 impaired glutamate uptake and glutamate utilization in hippocampal slices. **(A)** Metabolic mapping of [U-^13^C]glutamate in hippocampal slices of WT mice (black bars) and synGLT-1 KO mice (red bars). The slices were incubated in the presence of 200 μM [U-^13^C]glutamate in addition to 5 mM D-glucose (^12^C). Metabolism of ^13^C enriched substrates will lead to ^13^C incorporation in TCA cycle metabolites and derived amino acids, visualized by red circles (^13^C) and gray circles (^12^C). **(B)** Amino acid content in hippocampal slices of WT mice (black bars) and synGLT-1 KO mice (red bars) incubated with 200 μM [U-^13^C]glutamate in addition to 5 mM D-glucose. AAT, aspartate aminotransferase; GAD, glutamate decarboxylase; GDH, glutamate dehydrogenase. Mean ± SEM, *n* = 10, each point derived from individual animals, Student’s *t*-test with Benjamini-Hochberg correction, *p < 0.05. See text for exact p values.

To characterize the metabolic compromise present in the synGLT-1 KO slices that might contribute to excitotoxic injury, we performed metabolic labeling studies with ^13^C enriched glutamate ([U-^13^C]glutamate). By exposing slices to [U-^13^C]glutamate and then using mass spectrometry, we were able to measure the ^13^C label in intracellular glutamate as well as TCA cycle intermediates and derived amino acids (Figure 8A). We found a significant decrease in the ^13^C-labeling of intracellular glutamate [*t* = 4.212, *p* = 0.000524, *n* = 10 WT animals, *n* = 10 KO animals], consistent with the synapsin 1-Cre driven deletion of GLT-1 in neurons and previous studies of the impact of knockout of GLT-1 in neurons on uptake radiolabeled glutamate into synaptosomes (Petr et al., 2015; Rimmele and Rosenberg, 2016; Zhou et al., 2019; McNair et al., 2020). These studies establish that even though neuronal GLT-1 is a small fraction of total brain GLT-1, it is capable of actual transport of glutamate across the plasma membrane of axon terminals, and, in fact, mediates a disproportionately large fraction of uptake of glutamate into synaptosomes when assayed using radiolabeled substrate. A similar conclusion was reached in studies of D-aspartate uptake into hippocampal slices (Furness et al., 2008). As expected from a decrease in glutamate uptake in the synGLT-1 KO slices, ^13^C-labeling of malate [*t* = 2.287, *p* = 0.0345, *n* = 10 (WT), 10 (KO)], aspartate [*i* = 4.212, *p* = 0.000524, *n* = 10 (WT), 10 (KO)], citrate [*t* = 3.253, *p* = 0.00441, *n* = 10 (WT), 10 (KO)], α-ketoglutarate [*t* = 2.185, *p* = 0.0423, *n* = 10 (WT), 10 (KO)] and GABA [*t* = 2.231, *p* = 0.0386, *n* = 10 (WT), 10 (KO)] were likewise significantly decreased. Quantification of intracellular amino acids amounts in the slices (Figure 8B) showed decrease in glutamate [*t* = 2.547, *p* = 0.0202, *n* = 10 (WT), 10 (KO)] and aspartate concentrations [*t* = 2.833, *p* = 0.0110, *n* = 10 (WT), 10 (KO)] in the synGLT-1 KO slices, which is in line with previous observations. Taken together, the ultrastructural and metabolic labeling studies confirm that hippocampal slices of synGLT-1 KO mice display the same metabolic phenotype as observed *in vivo* (McNair et al., 2019) and are consistent with the hypothesis that metabolic perturbation caused by the deletion of GLT-1 from axon terminals could drive excitotoxicity in the synGLT-1 KO slices.

## Discussion

In this study, we found impairment of synaptic responses in the CA1 region of hippocampal slices from animals with a conditional knockout of GLT-1 in neurons. Field EPSPs were either absent or decreased in slices from 30 to 50 weeks as well as 20 weeks old animals but were not consistently diminished in slices from 10 weeks old animals. We compared the effect of conditional inactivation of GLT-1 in neurons with conditional inactivation of GLT-1 in astrocytes, and, remarkably, fEPSPs in slices from the astrocytic GLT-1 knockout at 40 weeks were not decreased, compared to wild-type littermates, suggesting an important role of neuronal GLT-1 in the recovery of synaptic function in the CA1 region of hippocampal slices.

CA3 neurons express GLT-1 mRNA at very high levels, perhaps the highest in the brain, whereas CA1 neurons express very low if any GLT-1 mRNA (Berger et al., 2005). Because of the high expression of GLT-1 in CA3 neurons, we expected that the CA3-CA1 synapse would be particularly relevant for studying the effects of deletion of GLT-1 in neurons on synaptic function and synaptic health. In other regions, the expression of GLT-1 in axon terminals might be significantly lower or non-existent, and in these regions, it might be expected that the phenotype we observed by recording in the stratum radiatum might not be present.

Previous studies have shown that GLT-1 is not significantly reduced in the synGLT-1 KO by immunoblot analysis of forebrain lysates (Petr et al., 2015) consistent with the small fraction (5–10%) of total GLT-1 expressed in axon terminals (Furness et al., 2008). Light microscopic (LM) and electron microscopic (EM) immunocytochemical studies focused on the hippocampus showed that at the LM level, there was no obvious loss of GLT-1 immunoreactivity in the hippocampus in the synGLT-1 KO in any region (Petr et al., 2015). At the ultrastructural (EM) level, in the same report, it was shown that there is a ca. 90% reduction in GLT-1 labeling of excitatory axon terminals in the stratum radiatum of synGLT-1 KO animals (Petr et al., 2015). In a subsequent study, it was shown that ^3^H-L-glutamate uptake is decreased 84% in crude synaptosomes prepared from the hippocampus of synGLT-1 KO animals compared with littermate controls (McNair et al., 2020), consistent with the expression of GLT-1 in axon terminals in the hippocampus (Chen et al., 2004; Furness et al., 2008) and the efficacious deletion of GLT-1 from axon terminals in this region by the use of synapse-Cre mediated recombination (Petr et al., 2015; Zhou et al., 2019). Zhou et al. (2019) also reported that GLT-1 immunoreactivity is not detectably altered at the LM level in the hippocampus in the synGLT-1 KO, but ^3^H-L-glutamate uptake into crude hippocampal synaptosomes is diminished.

We considered the possibility that persistent tissue damage produced by excitotoxic injury might be contributing to the impairment of functional recovery in the synGLT-1 KO slices. Kynurenic acid, which has been previously used in millimolar concentrations to prevent excitotoxic injury to brain slices during preparation (Mitra and Brownstone, 2012), provided partial functional restoration of function when present at 3 mM during the recovery period. Although the NMDA receptor antagonist D-APV (Paoletti and Neyton, 2007), either alone or together with the non-NMDA glutamate receptor antagonist DNQX (Honore et al., 1988; Sheardown et al., 1990), had no effect, MK801, a non-competitive antagonist (Huettner and Bean, 1988; Chen and Lipton, 1997) was completely protective. Extracellular glutamate scavenging, in the form of GPT plus pyruvate during the recovery period, promoted partial recovery of physiological function in the synGLT-1 KO slices, providing additional evidence that excitotoxic injury blocks recovery of function in the synGLT-1 KO slices. The lack of complete protection might be due to the absence of the scavenging system in the recording medium, or incomplete penetration of GPT and/or pyruvate into the depths of the slice. MK801, which is a non-competitive channel blocker whose washout is at least partially dependent on channel opening (McKay et al., 2013), might provide greater protection than APV because of incomplete washout during the recording period. Other examples of protection of hippocampal slices against excitotoxicity by MK801 but not APV have been reported (Schurr et al., 1995a, b; Schilp et al., 1999; Pringle et al., 2000), as well as a recent observation that MK801, but not APV, downregulates the expression of misfolded isoforms (PrP^Sc^) of cellular prior protein (PrP^C^; Zattoni et al., 2021).

Excitotoxicity as a cause of injury in acute slices was reported initially by Feig and Lipton (Feig and Lipton, 1990), and it has been invoked in many subsequent studies as a reason for using glutamate receptor antagonists during slice preparation to block injury and promote recovery (Buskila et al., 2020). Feig and Lipton showed that morphological evidence of injury in guinea pig slices, in particular swelling of neuronal cell bodies, was alleviated by inclusion of ketamine, an NMDA receptor blocker in the recovery medium, or using a medium lacking calcium but with high magnesium (10 mM). Interestingly, they found that the decline in ATP content in acute slices, which had been documented previously (Whittingham et al., 1984), was not affected by either ketamine or low calcium/high magnesium, and suggested that the energy collapse in acute slices might underlie increased vulnerability to excitotoxicity (Novelli et al., 1988; Henneberry et al., 1989a, b). The novelty of the present observations is in finding that heightened vulnerability to excitotoxicity distinguishes synGLT-1 KO slices from slices of *both* WT littermates and of gfapGLT-1 KO slices. This heightened vulnerability might be due to metabolic compromise, making CA1 neurons more vulnerable to the levels of glutamate normally encountered in acute slices, or to a disturbance of glutamate homeostasis in proximity to post-synaptic NMDA receptors, or to a change in NMDA receptor signaling, or a combination of these abnormalities.

Previous studies have examined the impact of constitutive deletion of GLT-1 in all cells, including both astrocytes and neurons, on synaptic transmission in the CA1 region of hippocampal slices (Tanaka et al., 1997). Of necessity, these studies were performed on animals significantly younger than those used in the present study (5–7 weeks) because GLT-1 pan KO mice have a 50% survival at 6 weeks on the original mixed background (Tanaka et al., 1997). Tanaka et al. (1997) found that the peak concentration of synaptically released glutamate was increased in the pan KO, consistent with an important role for GLT-1 in glutamate clearance from the synaptic cleft. In a subsequent study, NMDA receptor-dependent LTP was found to be impaired in GLT-1 pan KO mice due to excess basal activation of NMDA receptors, which could be rescued by a low concentration of D-APV (Katagiri et al., 2001). More recently, electrophysiological studies have been performed in slices from an astrocytic GLT-1 knockout (Aida et al., 2015) using animals at 12–16 weeks and showed normal synaptic transmission at corticostriate synapses in response to single stimuli, consistent with our observations related to the astrocytic GLT-1 KO reported here in the CA1 region of the hippocampus.

The observation in the present study that slices from synGLT-1 KO animals are functionally impaired due to excitotoxicity, whereas slices from gfapGLT-1 KO animals recover similarly to WT animals is surprising since the defect in clearance of glutamate is expected to be much greater in the astrocytic KO. It may be argued that glutamate transporters located in presynaptic terminals, because of their special localization, make an outsize contribution to glutamate clearance “where it counts” because they are close to the sites of the release of glutamate. However, this view is not consistent with our observations that stimulus-evoked glutamate accumulation was similar between genotypes. An alternative explanation is that the cause of the excitotoxic injury in the synGLT-1 KO slices is not a deficit in glutamate clearance *per se* but rather a metabolic defect related to the absence of GLT-1 in synaptic terminals resulting in increased vulnerability to excitotoxicity in postsynaptic cellular elements (spines, dendrites, cell bodies). Importantly, the impairment of synaptic utilization of glutamate reported previously in synaptosomal preparations from the synGLT-1 (McNair et al., 2019, 2020) was also observed in acute hippocampal slices of the synGLT-1 KO mice in the present studies.

One explanation to consider for the “normal” behavior of slices from gfapGLT-1 KO animals is that the astrocyte-specific knockout is not complete. In a previous publication characterizing the astrocyte-specific and neuron-specific GLT-1 knockouts, it was found that the astrocyte-specific knockout reduced GLT-1 expression by 75–95% (Petr et al., 2015). It is possible that the remainder of GLT-1 in the astrocyte KO is sufficient to provide protection. An observation that might be relevant to this question is that in the light microscopic immunocytochemistry for GLT-1 performed in the astrocytic GLT-1 KO in that study, there were certain cells in the neuropil that stained strongly for GLT-1, despite the KO (Petr et al., 2015). It is possible that these cells make a large contribution to the clearance of glutamate necessary for slice recovery.

The astrocytic GLT-1 KO was induced postnatally using an inducible driver of Cre-recombinase expression, whereas the neuronal GLT-1 KO was driven constitutively by a synapsin-Cre driver, suggesting the possibility that compensatory pathways activated in response to the two types of knockout might be different. However, no evidence was found for upregulation of GLAST, the other major glutamate transporter, in the astrocyte- or neuron-specific knockouts by immunoblot analysis (Petr et al., 2015). Even if residual glutamate clearance activity mediated by persistent expression of GLT-1 or other transporters contributes to slice recovery in the astrocytic GLT-1 KO, that would not explain why the neuronal knockout, which by immunoblot analysis does not delete enough GLT-1 to be readily detectable on immunoblot analysis (Petr et al., 2015), produces the impairment of recovery that it does, given that astrocytic glutamate clearance is still intact. The issue may not be glutamate clearance *per se* but some other factor, perhaps related to a metabolic or signaling function of GLT-1 expressed in axon terminals.

It is conceivable that the postnatal exposure to tamoxifen in some way provides the slices from adult astrocytic GLT-1 KO protection against the excitotoxic injury observed in the neuronal GLT-1 KO. However, such long-term protection has never been demonstrated. Instead, there is literature demonstrating an acute beneficial effect of tamoxifen in OGD in a brain slice model (Wakade et al., 2008), focal ischemia in the CNS (Zhang et al., 2005, 2007, 2009), and manganese toxicity (Lee et al., 2009a, b; Pajarillo et al., 2018). In our studies, the tamoxifen is administered to gfapGLT-1 KO pups at least 9–10 weeks prior to the age when slices are taken for experiments. It is unlikely that the normal recovery of gfapGLT-1 KO slices is due to an enduring effect of tamoxifen exposure. In any case, even if that were true, the unexpected, heightened vulnerability of synGLT-1 KO slices to excitotoxicity would not be explained.

The “WT controls” used in most experiments in this study are flox controls (GLT-1^flox/flox^) from the same litter. Conceivably insertion of loxP cassettes has an effect on gene expression and function. Breeding to produce Cre negative and flox negative (GLT-1^+/+^) littermates of animals to serve as controls for test animals that are homozygous floxed and expressing Cre (GLT-1^flox/flox^; Cre-recombinase+) is very inefficient, because of low yields. The breeding scheme we have chosen produces 50% test animals and 50% animals used as littermate controls and allows for the testing of the effects of Cre-mediated excision on a constant genetic background (GLT-1^flox/flox^). This approach requires additional experiments to test the effects of Cre recombinase expression itself, which we have done testing slices from wild-type animals (GLT-1^+/+^) compared with synapsin-Cre expressing animals on a wild-type background (GLT-1^+/+^;Syn-Cre; SynCre+ vs. SynCre- in Figure 2E). Of note, there is not a significant difference between the fEPSP generation in slices from GLT-1^flox/flox^ animals (Figures 1, 2, 5, 6) and GLT-1^+/+^ animals (Syn-Cre- in Figure 2).

GLT-1 has been shown to physically interact with multiple mitochondrial proteins as well as enzymes involved in glycolysis, presumably by one or more scaffolding proteins that have yet to be identified (Genda et al., 2011). It has been suggested that this association between GLT-1 and proteins involved in energy production serves the function of localizing energy production close to sites of GLT-1 mediated transport, which is highly energy consuming, and possibly to provide glutamate as a fuel for mitochondrial metabolism (Robinson et al., 2020). The assumption has been that this association takes place in astrocytes, but recent data (McNair et al., 2019, 2020) and the present studies raise the question of whether these associations are taking place in neurons. This question has, as yet, not been directly addressed.

In two previous studies (McNair et al., 2019, 2020), we analyzed the prevalence and cristae density of mitochondria in axon terminals, because morphological differences such as cristae density are reflective of the efficiency of mitochondrial metabolism, including ATP production (Leveille et al., 2017). As was observed for the hippocampus at 8–10 weeks of age (McNair et al., 2019), the hippocampus of animals at 22–24 weeks of age in the present study revealed a decrease in the inter-cristae distance within presynaptic mitochondria for the synGLT-1 KO animals, relative to WT littermates (Figure 7E). The inter-cristae distance may have been influenced not only by the genotype but also by the age: the measured values were less for both genotypes at 22–24 weeks of age, compared to the distances observed at 8–10 weeks of age. However, since the tissues of the two age groups were not processed jointly for electron microscopic analysis, we cannot rule out the possibility that the age difference was due to unintentional differences in tissue preparation for electron microscopy. The results suggest that the loss of GLT-1 within axon terminals may be compensated by increased efficiency of the mitochondrial TCA cycle and ATP production (Gomes et al., 2011; Cogliati et al., 2013; Leveille et al., 2017; McNair et al., 2019).

In contrast to the previous study in which we analyzed the impact of neuronal GLT-1 KO upon the dorsal hippocampus at 8–10 weeks of age (McNair et al., 2020), neuronal GLT-1 KO at 22–24 weeks of age (this study) no longer resulted in an increase of the presynaptic mitochondrial frequency, relative to the frequency measured in the CA1 region of the hippocampus of WT littermates (Figure 7D). The difference observed in mitochondrial frequency in axon terminals in the synGLT-1 KO at 20 weeks and 10 weeks in comparison with control littermates is unlikely to have resulted from subtle unintentional differences in tissue processing because differences in tissue handling could not have caused differential disappearance or appearance of axons or of mitochondria postmortem or during transcardial perfusion of animals to fix brain tissue. The difference across the ages could reflect developing compensation for the metabolic defect known to occur in the synGLT-1 KO mice. Early on, before compensation for this defect is fully developed, more mitochondria might be trafficked to the terminals, whereas at older ages, some form of metabolic compensation may have occurred, so that the mitochondria traffic into the terminals does not have to be increased. One could explain, potentially, the age dependence of the phenomenon of increased vulnerability to excitotoxicity of slices from the synGLT-1 KO at 20 weeks of age compared with 8–10 weeks of age by the relative decrease in mitochondria in the terminals at 20 weeks of age compared with 10 weeks of age. The increased density of mitochondria in terminals at 10 weeks may allow the slices to cope with the insult of slice preparation more readily than they are able to at 20 weeks of age.

The present study implicates neuronal GLT-1 in regulating the vulnerability of neurons in the CNS to excitotoxicity, which has long been thought to play an important role in acute and chronic neurodegenerative disorders (Lewerenz and Maher, 2015; Choi, 2020). The work presented here suggests that the hippocampal slice preparation provides a useful model system for the study of the metabolic role of GLT-1 expressed in neurons and the consequences of interfering with it. The importance of neuronal GLT-1 in regulating the vulnerability of CA1 neurons to excitotoxicity in hippocampal slices raises the possibility that there are pathways that have been little explored that may play a determinative role in devastating neurodegenerative disorders and that need to be better understood.

## Data Availability Statement

The raw data supporting the conclusions of this article will be made available by the authors, without undue reservation.

## Ethics Statement

The animal study was reviewed and approved by Children’s Hospital Boston Institutional Animal Care and Use Committee.

## Author Contributions

TR, SL, JA, AR, BA, DS, CA, CD, and PR contributed to the design of experiments and analysis and interpretation of data. PR, TR, CD, JA, BA, and CA wrote the manuscript. PR, TR, CD, JA, BA, CA, and DS edited and revised the manuscript. TR, SL, CA, JA, EW, and JW performed experiments.

## Conflict of Interest

DS is a director and consultant of Prothena Biosciences. The remaining authors declare that the research was conducted in the absence of any commercial or financial relationships that could be construed as a potential conflict of interest.

## Publisher’s Note

All claims expressed in this article are solely those of the authors and do not necessarily represent those of their affiliated organizations, or those of the publisher, the editors and the reviewers. Any product that may be evaluated in this article, or claim that may be made by its manufacturer, is not guaranteed or endorsed by the publisher.
